# Cancer Therapy Approval Timings, Review Speed, and Publication of Pivotal Registration Trials in the US and Europe, 2010-2019

**DOI:** 10.1001/jamanetworkopen.2022.16183

**Published:** 2022-06-10

**Authors:** Mark P. Lythgoe, Aakash Desai, Bishal Gyawali, Philip Savage, Jonathan Krell, Jeremy L. Warner, Ali Raza Khaki

**Affiliations:** 1 Department of Surgery and Cancer, Imperial College London, London, United Kingdom; 2 Division of Medical Oncology, Mayo Clinic, Rochester, Minnesota; 3 Division of Cancer Care and Epidemiology, Departments of Oncology and Public Health Sciences, Queen’s University Cancer Research Institute, Kingston, Canada; 4 Brighton and Sussex University Hospitals NHS Trust, Brighton, United Kingdom; 5 Division of Hematology and Oncology, Vanderbilt University, Nashville, Tennessee; 6 Division of Oncology, Department of Medicine, Stanford University School of Medicine, Palo Alto, California

## Abstract

**Importance:**

Ensuring patients have access to safe and efficacious medicines in a timely manner is an essential goal for regulatory agencies, one which has particular importance in oncology because of the substantial unmet need for new therapies. The 2 largest regulatory agencies, the US Food and Drug Administration (FDA) and the European Medicines Agency (EMA), have pivotal global roles, and their recommendations and approvals are frequently followed by other national regulators.

**Objective:**

To compare market authorization dates for new oncology therapies approved in the US and Europe over the past decade and to examine and contrast the regulatory activities of the FDA and EMA in the approval of new cancer medicines.

**Design, Setting, and Participants:**

This cross-sectional study reviewed the FDA and EMA regulatory databases to identify new oncology therapies approved in both the US and Europe from 2010 to 2019, and characterization of the timings of regulatory activities. Statistical analysis was performed from January to April 2022.

**Main Outcomes and Measures:**

Regulatory approval date, review time, submission of market authorization application, accelerated approval or conditional marketing authorization status and proportion of approvals prior to peer-reviewed publication of pivotal trial results.

**Results:**

In total, 89 new concomitant oncology therapies were approved in the US and Europe from 2010 to 2019. The FDA approved 85 oncology therapies (95%) before European authorization and 4 therapies (5%) after. The median (IQR) delay in market authorization for new oncology therapies in Europe was 241 (150-370) days compared with the US. The median (IQR) review time was 200 (155-277) days for the FDA and 426 (358-480) days for the EMA. Sixty-four new licensing applications (72%) were submitted to the FDA first, compared with 21 (23%) to the EMA. Thirty-five oncology therapies (39%) were approved by the FDA prior to pivotal study publication, whereas only 8 (9%) by the EMA.

**Conclusion and Relevance:**

In this cross-sectional study, new oncology therapies were approved earlier in the US than Europe. The FDA received licensing applications sooner and had shorter review times. However, more therapies were approved prior to licensing study publication, leaving uncertainty for practitioners regarding clinical utility and safety of newly approved therapies.

## Key Points

**Question:**

When a new cancer therapy is approved by both the US Food and Drug Administration (FDA) and the European Medicines Agency (EMA), are there notable differences in approval timings and review speed?

**Findings:**

This cross-sectional study found that of 89 new oncology therapies approved by both the FDA and EMA from 2010 to 2019, the FDA approved 95% of therapies first, with a median delay to market authorization in Europe of 241 days.

**Meaning:**

The findings of this study suggest that patients in the US have access to new oncology therapies earlier than in Europe.

## Teaser

This cross-sectional study compares market authorization dates for new oncology therapies approved in the US and Europe from 2010 to 2019 and examines and contrasts the regulatory activities in the approval of new cancer medicines.

## Introduction

Market authorization of new therapies granted by regulatory agencies require evidence of safety and therapeutic efficacy based on adequate and well controlled studies. The 2 largest global regulators are the US Food and Drug Administration (FDA) and the European Medicines Agency (EMA).^1^ As such, they frequently set industry standards and guidance, routinely followed by other national regulatory agencies.^2^

The past decade has witnessed a record number of new oncology therapy approvals, including many first-in-class or breakthrough therapies, requiring timely review and authorization from regulatory agencies to provide prompt access to patients in need.^3,4,5^ Over this same period, new review pathways have been developed by both the FDA (Breakthrough Designation) and EMA (Priority Medicines; PRIME) to enhance support for the development and review of medicines to treat serious conditions. Furthermore, expedited approval pathways (accelerated approval by the FDA and conditional marketing authorization by the EMA) have also been used to address many areas of unmet need within oncology. These approvals are made with less comprehensive clinical data but with the expectation for further data before granting regular approval.^6^

The activities of the FDA and EMA are frequently compared, particularly in reference to approval times for new therapy and device registrations.^1,7,8,9^ Despite differences in approval processes, prior studies have shown a close alignment between FDA and EMA in more than 90% of new therapy registrations across all therapeutic areas.^1^ However, no recent comparisons of the regulatory activities of the FDA and EMA in the approval of new oncology therapies have been conducted. A study by Roberts and colleagues^10^ found that market authorization of new oncology therapies between 2003 and2010 was 238 days later in Europe compared with the US. However, it is unknown how expedited review, approval initiatives, and the challenge of new therapeutic classes over the past decade has affected review speed and approval timings between the FDA and EMA.

We therefore investigated new oncology therapies authorized by each regulator over the past decade, comparing initial market authorization date and characterizing submission dates, review length, and approval timings relative to publication of pivotal clinical studies supporting regulatory authorization.

## Methods

This cross-sectional study did not involve individual patient information; involving only publicly available clinical trial and medicine regulator data, which has been previously reported. Therefore, this study was deemed to not require institutional review board approval, according to 45 CFR §46. This study followed the Strengthening the Reporting of Observational Studies in Epidemiology (STROBE) reporting guideline.

The primary goal of this study was to compare market authorization dates for new oncology therapies approved both in the US and Europe. We also evaluated submission dates, review length, and approval relative to publication of the supporting clinical trial(s) in a peer-reviewed journal. We identified new cancer therapies approved by both the FDA and EMA from January 1, 2010, to December 31, 2019, by a review of each medicine regulator’s website, performed on April 1, 2021.^11,12^ To ensure all new therapies approved by both agencies were captured, we included any new oncology therapy approved within the 10-year period by either agency. Only initial approvals for new cancer therapies were considered, any supplementary licenses (indications beyond the initial license), supportive medicines, and biosimilars were excluded from analysis. New formulations of therapies with an existing market authorization were also excluded. We adopted similar methodology to prior studies to facilitate comparison of overall temporal trends in regulatory activities.^10,13^

Data sources included approval letters, prescribing information, and review documents from both the EMA and FDA regulatory databases.^11,12^ For each new therapy approved in the US, we collected the submission date for the new drug application (NDA) or biologics license application (BLA), the date of market authorization and if accelerated approval had been granted. New therapy approval in the European Union requires 2 steps to allow market authorization. The EMA’s committee for medicinal products for human use (CHMP) must first issue a positive opinion followed by European Commission adoptionn to allow market authorization in all European Union (EU) member countries. Therefore, for each new therapy, we collected the EMA submission date, positive CHMP opinion date, and formal European Commission (EU market authorization) adoption date. We also identified if drugs had received conditional authorization status by the EMA (akin to FDA accelerated approval program) with requirements for subsequent confirmatory data to be provided before full standard authorization can be granted. Further information on therapeutic classification, initial licensing indication, and pivotal trials used for efficacy evaluation were also recorded from each regulator’s database.

### Statistical Analysis

We compared the date of market authorization for new anticancer therapies in the US and Europe, calculating the median difference between FDA approval and the European Commission adoption date in calendar days (eTable 1 in the Supplement). To evaluate regulatory review speed, we calculated the time interval, in calendar days, between the regulatory submission and market authorization dates, calculating the median review duration for each agency. Furthermore, for new therapies approved in the EU, the date of positive CHMP and European Commission adoption were identified, and the median time interval between regulatory submission and European Commission adoption calculated. Subgroup analysis of therapeutic groups by mechanism of action was performed using therapeutic classifications used by the FDA and previously established in similar analyses.^14^

The timings of pivotal clinical trial publications relative to market authorization date in the US and Europe were also compared. The date of first publication (online publication) of the registrational trial associated with therapy approval was identified by searching PubMed or ClinicalTrials.gov records. The proportion of therapy approvals in Europe and the US that preceded publication, and the median time from therapy authorization to publication date (a positive number indicated the publication came after authorization and a negative number that publication preceded authorization) were calculated. Statistical analysis was performed from January to April 2022 using Microsoft Excel version 16.6 (Microsoft Corp), R statistical software version 3.6.2 (R Project for Statistical Computing) and Stata version 16 (StataCorp).

## Results

**Figure 1.**
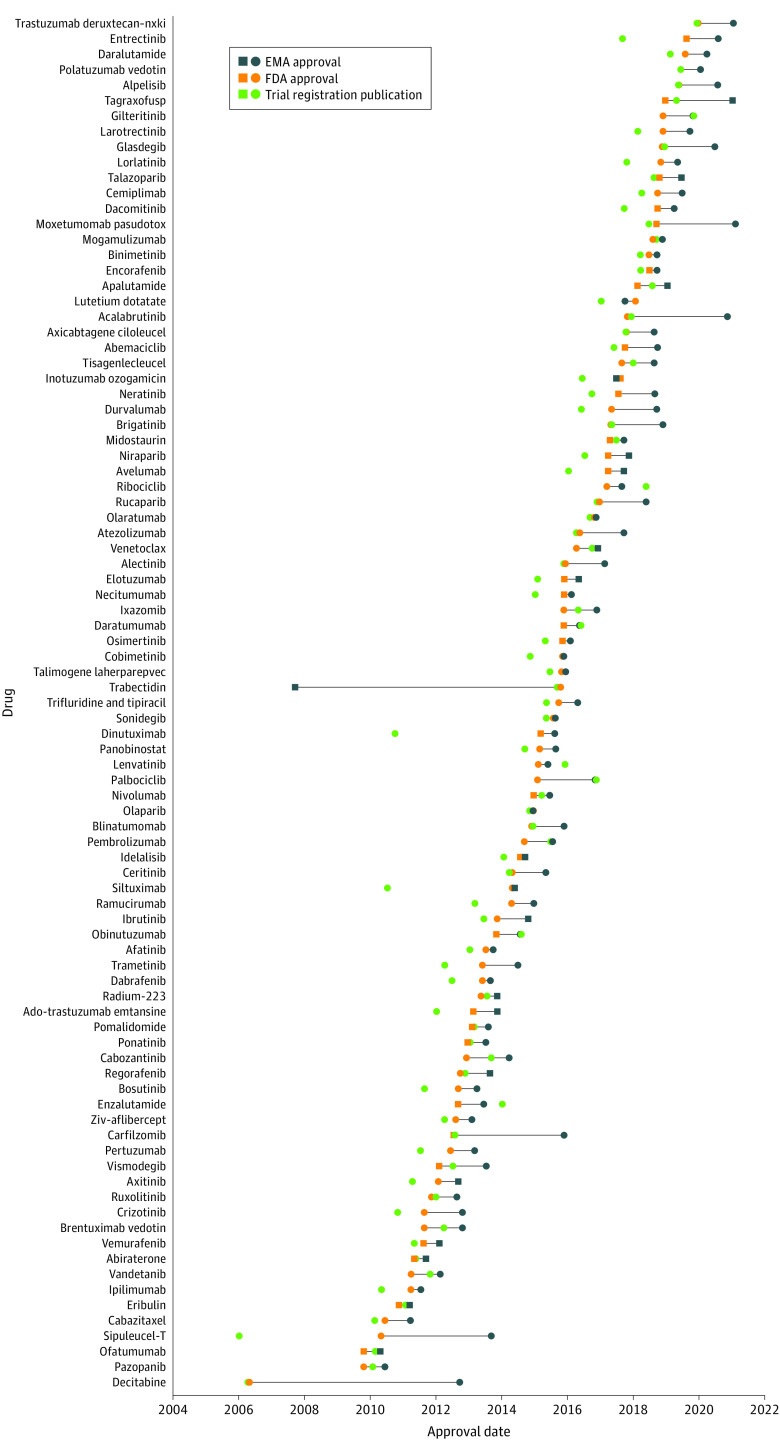
Timing of New Oncology Drug Approval by the US Food and Drug Administration (FDA) and the European Medicines Agency (EMA) Timing of FDA and EMA approval relative to each other and publication of registrational clinical trial. Expedited approvals (eg, FDA accelerated approval or EMA conditional marketing approval) are depicted with squares, and standard approval with circles.

From January 1, 2010, until December 31, 2019, 89 new oncology therapies were approved in both the US and Europe (Figure 1, eTable 1 in the Supplement). The FDA approved new oncology therapies a median (IQR) of 241 (150-370) days before European market authorization. The FDA approved 85 oncology (95%) therapies before European authorization and 4 therapies (5%) after. The 4 drugs approved in Europe before the US were olaparib (3 days earlier), inotuzumab ozogamicin (50 days earlier), lutetium Lu-177 dotatate (122 days earlier), and trabectedin (2958 days earlier).

### Submission Date

There were 64 new licensing applications (72%) submitted first to the FDA, compared with 21 (23%) to the EMA and 4 (5%) on the same day to both regulators. The FDA received new drug licensing applications a median (IQR) of 20 (0-98) days earlier than the EMA. All 4 therapies (5%) authorized for use in Europe first had earlier market authorization applications (including resubmissions) submitted to the EMA by a median (range) of 201 (2-3042) days. There were 12 (13%) therapies (acalabrutinib, carfilzomib, decitabine, durvalumab, gilteritinib, glasdegib, moxetumomab pasudotox, palbociclib, sipuleucel-T, tagraxofusp, tisagenlecleucel and trastuzumab deruxtecan) approved for use by the FDA before submission of the market authorization application to the EMA.

### Review Time

The median (IQR) review time from submission to approval for all therapies was 200 (155-277) days for the FDA and 426 (358-480) days for the EMA. As part of the 2-stage process for European Approval, submission to CHMP review was a median (IQR) of 363 (292-416) days followed by an additional median (IQR) of 62 (56-68) days before European Commission adoption and EU market authorization. The mean difference (SD) in regulatory review duration across all therapies by the FDA and EMA was 208 (116) days. Regulatory review was shorter by the FDA for all therapies, except lutetium dotatate Lu-177 resubmission (approval time 120 days shorter for EMA).

For initial approvals, the FDA issued 31 accelerated approvals (35%) and the EMA issued 23 conditional marketing authorizations (26%) (eTable 1 in the Supplement). Of these, 15 therapies (19%) were granted concurrent FDA accelerated approval and EMA conditional marketing authorization. The median (IQR) FDA review time for new therapies granted accelerated approval was 179 (143-253) days, 31 days shorter than regular authorization. The median (IQR) EMA review time for new therapies granted conditional marketing authorization was 476 (396-523) days, 56 days longer than standard authorization.

Of the 31 initial approvals receiving accelerated approval from the FDA, 19 therapies (61%) were subsequently granted regular approval, 3 drugs (16%) were withdrawn and 9 (27%) retain this status, awaiting further information from confirmatory trials. For the 23 therapies granted conditional marketing authorization by the EMA, 10 (43%) have been converted to regular approval, 1 (4%) was withdrawn and 12 (52%) continue with this status.

### Therapeutic Class

**Figure 2.**
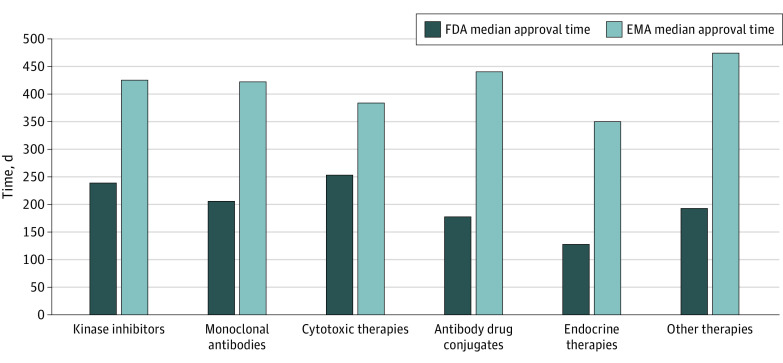
Median Approval Time in Days by Regulator for Anticancer Therapies by Therapeutic Class EMA indicates European Medicines Agency; FDA, US Food and Drug Administration.

The distribution of mechanism of action for the oncology approvals in our study were: 36 kinase inhibitors, 20 monoclonal antibodies, 4 cytotoxic therapies, 6 antibody-drug conjugates, 4 hormonal therapies, and 19 other therapies (eTable 2 in the Supplement). Approval times by therapeutic class are shown in Figure 2. All cumulative therapy subtypes had a shorter review time by the FDA compared with the EMA. Both the FDA and EMA had the shortest median (range) review time for endocrine therapies (FDA: 128 [102-154] days; EMA: 350 [262-386] days), whereas the longest for the FDA was cytotoxic therapies (254 [78-333] days), and the longest for the EMA were antibody-drug conjugates (441 [range 241-513] days) and other therapies (475 [range 293-731] days). The smallest differences in review times between medicine regulators was 131 days for cytotoxic therapies and the longest was 264 days for antibody-drug conjugates and 282 days for other therapies (eTable 2 in the Supplement).

### Publication Timing

**Figure 3.**
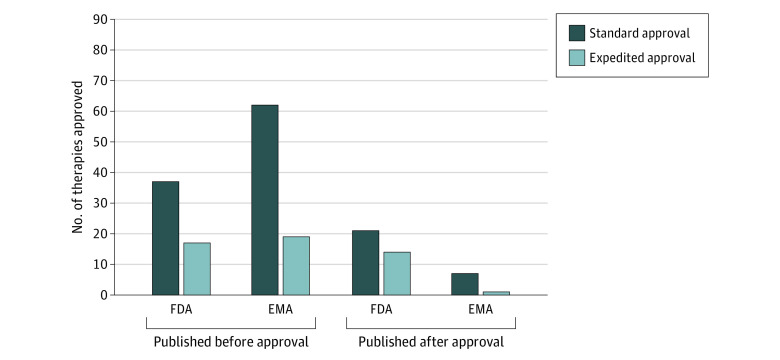
Comparison of Drugs Approved by the US Food and Drug Administration (FDA) and the European Medicines Agency (EMA) Before and After Publication of Pivotal Trial Results

We also evaluated the timing of study publication relative to drug approval; overall, 35 drugs (39%) were approved by the FDA before publication of the pivotal trial, compared with 8 drugs (9%) approved by the EMA (Figure 3). The median (IQR) time from drug approval to publication was −45 (−288 to 56) days by the FDA and −344 (−554 to −149) days by the EMA. Among the 35 drugs approved by the FDA prior to publication, the median (IQR) time from approval to publication was 125 (31 to 220) days; and among the 8 drugs approved by the EMA prior to publication, the median (IQR) time to publication was 100 (9 to 237) days. The eFigures 1 and 2 in the Supplement show waterfall plots of timing (in days) of each drug approval relative to study publication by the FDA and EMA, respectively.

## Discussion

This cross-sectional study found that 95% of new oncology therapies were approved in the US before receiving European market authorization. In addition, we found that most pharmaceutical companies submit new market authorization to the FDA before the EMA, and the FDA review time was half that of the EMA. However, shorter review times and earlier approval by the FDA mean that more drugs are withdrawn, 3 drugs by the FDA and 1 drug by the EMA, from the market and approximately one-third of medicines are approved before registrational study publication, compared with less than one-in-ten for new EMA oncology approvals.

Between 2003 and 2010, all new oncology therapies were approved earlier by the FDA, with a median delay of 238 days between FDA approval and European Commission adoption.^10^ We found that this delay between market authorizations continues to persist and has increased slightly to 241 days. However, unlike in previous studies, a small number of therapies, such as olaparib, were approved in Europe before receiving FDA approval. The number of concordant new oncology approvals by both regulators over the past decade has risen substantially compared with earlier studies^10^ and include several new first-in-class therapies which may have compounded efforts to reduce review times.^7^ Notably, both lutetium Lu-177 dotatate and trabectedin required NDA resubmissions to the FDA following initial unsuccessful applications, likely resulting in earlier EMA approval. These findings add to the growing literature that shows the FDA, in oncology and beyond, consistently approves new therapies more rapidly than the EMA and other medical regulators.^1,8,10^

Oncology therapy development is distinctive, receiving the highest proportions of expedited approvals by the FDA (eg, priority review, fast-track, and accelerated approval) and EMA (eg, accelerated assessment and conditional marketing authorization).^14,15,16^ Both FDA accelerated approval and EMA conditional marketing authorization support earlier approvals of medicines which address unmet medical needs based on surrogate end points that are expected to predict clinical efficacy in later confirmatory trials. Conditional marketing authorization in Europe is valid for 1 year and renewed annually to ensure market authorization holders are fulfilling specific obligations within defined timelines. Accelerated approval by the FDA is not subject to annual review, however, confirmatory trials must be completed within a specific timeframe. Although, many confirmatory studies are not completed until more than 3 years after initial approval.^17^ Over the past decade, each regulator has introduced additional programs, such as breakthrough designations (FDA) and PRIME (EMA), to further expedite drug development. Despite these initiatives, the median review time at both regulators for new oncology therapies has increased ( (by 14 days at the FDA and by 74 days at the EMA) compared with 2003 to 2010.^10^ The FDA accelerated approval reduces median review time by 31 days, whereas EMA conditional marketing authorization increases median review time by 60 days, suggesting differing regulatory approaches to market authorization of new therapies lacking definitive clinical outcome data. The delay between European Commission adoption and positive CHMP opinion has remained relatively unchanged.

We found that drug manufacturers continue to submit regulatory documents earlier to the FDA than the EMA. This may be partly driven by the greater market share and higher cost of new oncology therapies in the US compared with Europe.^18,19^ Notably, although 72% of manufacturers submitted new market authorization applications to the FDA before the EMA, this has fallen slightly from 80% reported between 2003 and 2010.^10^ Submissions to the EMA and FDA are typically based on the same clinical trials, however, submission to the EMA contained more complete and less preliminary data due to later submission times.^1^

An important consequence of early approval of therapies is uncertainty regarding appropriate use and overall benefit. For example, if a drug is approved prior to publication of the pivotal registration clinical trial, clinicians do not have complete information regarding the overall safety and efficacy. This can potentially put patients at risk in terms of patient selection for therapy and toxicity management. Furthermore, drugs approved using expedited approval pathways may be approved without randomized data and/or the use of surrogate end points, therefore leaving uncertainty regarding overall clinical benefit. The relative concordance in therapies approved by both regulators using expedited pathways suggests similarities in market authorization applications. However, the lower number of new oncology therapies receiving conditional marketing authorization and higher median review times suggests the EMA may have a more cautious approach to use. Furthermore, some drugs approved by the accelerated approval pathway have been subsequently pulled from the market after the confirmatory trial failed to show benefit (eg, durvalumab), and others have shown that confirmatory trials are often delayed and sometimes not completed at all.^17,20^

The balance between shortening approval times, allowing oncology patients to receive therapies earlier, and ensuring treatments are safe and efficacious is delicate. Concerns about the efficacy of oncology therapies authorized by accelerated review and surrogate end points have been raised as only 20% of drugs approved with a surrogate end point later showed improvement in overall survival.^21^ In addition, previous studies have shown that drugs that receive accelerated approval by the FDA are twice as likely to receive a black box warning (defined as known serious risk) or withdrawn from market.^22,23^

Despite differing review processes, there is considerable overlap and potential synergy to be gained by harmonizing therapy approval among the FDA, EMA, and other medicine regulators.^1^ The FDA participates in Project Orbis, a global collaborative review to facilitate faster patient access to innovative cancer therapies across multiple countries. This allows for concurrent submission, review, and regulatory action among partner countries (eg, Health Canada) and has approved several new oncology therapies.^24^ However, this project does not currently include the EMA.

### Limitations

This study has several limitations that merit discussion. Our analysis was limited to initial approvals; supplementary approvals constitute a sizable proportion of new oncology therapeutic approvals, and a further comparison would be informative. However, to compare with previous studies we chose to focus on initial market authorizations only. Similarly, we chose to exclude supportive medicines, biosimilars, new formulations, and supportive and ancillary drugs. Prior studies have also shown that subsequent approvals are more likely to have published data prior to regulatory approval, likely because once a drug is approved, disseminating clinical efficacy may change practice prior to regulatory approval.^25,26^

The primary focus of this study was the availability of new anticancer therapies in the US and Europe. We only considered official review times and did not take account of presubmission enquires or real-time oncology reviews used by both regulators as these are often confidential and information is frequently not in the public domain. New cancer therapies may have been initially approved by each regulator for differing cancer indication (eTable 1 in the Supplement), for example durvalumab was initially approved by the FDA for urothelial cancer and the EMA for non–small cell lung cancer. Furthermore, we did not include medical devices related to oncology (eg, imaging reagents, equipment and in vitro diagnostics) due to differences in regulatory approval processes and to ensure consistency with prior studies. We also did not consider date of submission for publication of pivotal registration study, as it is possible publication may have been delayed for reasons outside of the sponsor’s control (eg, editorial priority).

## Conclusions

In this cross-sectional study we found that new oncology therapies receive market authorization in the US earlier than in Europe. The FDA has shorter review times than the EMA overall and across all oncology therapy subtypes. Pharmaceutical manufacturers continue to submit market authorization applications earlier to the FDA, despite longer review times at the EMA. However, earlier FDA approvals come with the consequence that one-third precede publication of registrational trial, compared with only approximately 10% preceding European approval.

The past decade has seen record numbers of new oncology therapy authorizations in the US and Europe. It is critical that medicine regulators undertake rigorous scientific review of all new therapies to ensure efficacy, safety, and public confidence in cancer medicines.

## Notes

Supplement. **eTable 1.** New Oncology Drug Approvals by the FDA and EMA from 2010 to 2019 **eTable 2.** Therapies Classed as Other by Mechanism of Action **eFigure 1.** Waterfall Plot Showing Time Difference (in Days) Between FDA New Oncology Approval and Publication of Pivotal Trial Results **eFigure 2.** Waterfall Plot Showing Time Difference (in Days) Between EMA New Oncology Approval and Publication of Pivotal Trial Results
