# Supplementary material for: Cancer Therapy Approval Timings, Review Speed, and Publication of Pivotal Registration Trials in the US and Europe, 2010-2019
Source: JAMA Netw Open. 2022 Jun 10;5(6):e2216183. doi: 10.1001/jamanetworkopen.2022.16183 (PMC9187952; doi:10.1001/jamanetworkopen.2022.16183)
Supplement: Supplement. — eTable 1. New Oncology Drug Approvals by the FDA and EMA from 2010 to 2019 eTable 2. Therapies Classed as Other by Mechanism of Action eFigure 1. Waterfall Plot Showing Time Difference (in Days) Between FDA New Oncology Approval and Publication of Pivotal Trial Results eFigure 2. Waterfall Plot Showing Time Difference (in Days) Between EMA New Oncology Approval and Publication of Pivotal Trial Results [file jamanetwopen-e2216183-s001.pdf]

Supplemental Online Content

Lythgoe MP, Desai A, Gyawali B, et al. Cancer therapy approval timings, review speed, and publication of pivotal registration trials in the US and Europe, 2010-2019. *JAMA Netw Open.* 2022;5(6):e2216183. doi:10.1001/jamanetworkopen.2022.16183

eTable 1. New Oncology Drug Approvals by the FDA and EMA from 2010 to 2019

eTable 2. Therapies Classed as Other by Mechanism of Action

eFigure 1. Waterfall Plot Showing Time Difference (in Days) Between FDA New Oncology Approval and Publication of Pivotal Trial Results

eFigure 2. Waterfall Plot Showing Time Difference (in Days) Between EMA New Oncology Approval and Publication of Pivotal Trial Results

This supplemental material has been provided by the authors to give readers additional information about their work.

© 2022 Lythgoe MP et al. *JAMA Network Open.*

eTable 1: Concordant New Therapy Approvals by the US FDA and EMA

**Name**	**Initial Disease Indication**	**Treatment Type**	**US Food and Drug Administration (FDA)**			**European Medicines Agency (EMA)**			**First Approved by Medicine Regulator**	**Days between FDA and EMA market authorization**
			Submission date	Authorization Date	Time for review (days)	Submission date	Authorization Date	Time for review (days)		
Abemaciclib	Breast Cancer	KI	05/05/2017	09/28/2017	146	07/27/2017	09/26/2018	426	FDA	363
Abiraterone	Prostate Cancer	Endo	12/20/2010	04/28/2011	128	12/17/2010	09/05/2011	262	FDA	130
Acalabrutinib	Differing Indications	KI	06/13/2017	10/31/2017	140[AA]	10/14/2019	11/05/2020	388	FDA	1101
Ado-trastuzumab emtansine	Breast Cancer	ADC	08/27/2012	02/22/2013	179	08/30/2012	11/15/2013	442	FDA	266
Afatinib	Non-Small Cell Lung Cancer	KI	11/15/2012	07/12/2013	239	08/28/2012	09/25/2013	393	FDA	75
Alectinib	Non-Small Cell Lung Cancer	KI	06/07/2015	12/11/2015	187[AA]	09/08/2015	02/16/2017	527[CA]	FDA	433
Alpelisib	Breast cancer	KI	12/18/2018	05/24/2019	157	12/19/2018	07/27/2020	586	FDA	430
Apalutamide	Prostate Cancer	Endo	10/10/2017	02/14/2018	127	02/08/2018	01/14/2019	340	FDA	334
Atezolizumab	Urothelial Cancer	Mab	01/12/2016	05/18/2016	127[AA]	04/20/2016	09/20/2017	518	FDA	490
Avelumab	Merkel Cell Carcinoma	Mab	07/06/2016	03/23/2017	260[AA]	10/06/2016	09/18/2017	347	FDA	179

© 2022 Lythgoe MP et al. *JAMA Network Open.*

Axicabtagene ciloleucel	Diffuse Large B-cell Lymphoma	Other	03/31/2017	10/18/2017	201	07/29/2017	08/23/2018	390	FDA	309
Axitinib	Renal Cell Carcinoma	KI	04/14/2011	01/27/2012	288	04/19/2011	09/03/2012	503	FDA	220
Binimetinib	Melanoma	KI	06/30/2017	06/27/2018	362	07/28/2017	09/20/2018	419	FDA	85
Blinatumomab	Acute Lymphocytic Leukemia	Mab	09/19/2014	12/03/2014	75[AA]	10/09/2014	11/23/2015	410[CA]	FDA	355
Bosutinib	Chronic Myelogenous Leukaemia	KI	11/17/2011	09/04/2012	292	07/28/2011	03/27/2013	608[CA]	FDA	204
Brentuximab vedotin	Hodgkin & Anaplastic Large Cell Lymphoma	ADC	02/28/2011	08/19/2011	172[AA]	05/31/2011	10/25/2012	513[CA]	FDA	433
Brigatinib	Non-Small Cell Lung Cancer	KI	08/29/2016	04/28/2017	242[AA]	02/03/2017	11/22/2018	657	FDA	573
Cabazitaxel	Prostate Cancer	CC	03/31/2010	06/17/2010	78	04/20/2010	03/17/2011	331	FDA	273
Cabozantinib	Medullary Thyroid Cancer	KI	05/29/2012	11/29/2012	184	10/29/2012	03/21/2014	508[CA]	FDA	477
Carfilzomib	Multiple Myeloma	Other	09/27/2011	07/20/2012	297[AA]	01/22/2015	11/19/2015	301	FDA	1217
Cemiplimab	Squamous Cell Carcinoma	Mab	02/28/2018	09/28/2018	212	03/06/2018	06/28/2019	479[CA]	FDA	273

© 2022 Lythgoe MP et al. *JAMA Network Open.*

Ceritinib	Non-Small Cell Lung Cancer	KI	12/24/2013	04/29/2014	126[AA]	03/04/2014	05/06/2015	428[CA]	FDA	372
Cobimetinib	Melanoma	KI	12/11/2014	11/10/2015	334	09/02/2014	11/20/2015	444	FDA	10
Crizotinib	Non-Small Cell Lung Cancer	KI	03/30/2011	08/26/2011	149[AA]	07/28/2011	10/23/2012	453[CA]	FDA	424
Dabrafenib	Melanoma	KI	07/30/2012	05/29/2013	303	07/24/2012	08/26/2013	398	FDA	89
Dacomitinib	Non-Small Cell Lung Cancer	KI	01/31/2018	09/27/2018	239	02/09/2018	04/02/2019	417	FDA	187
Daratumumab	Multiple Myeloma	Mab	07/09/2015	11/16/2015	130[AA]	09/09/2015	05/20/2016	254[CA]	FDA	186
Darolutamide	Prostate Cancer	Endo	02/26/2019	07/30/2019	154	03/07/2019	03/27/2020	386	FDA	241
Decitabine	Myelodysplastic syndrome	Other	11/15/2005	05/02/2006	168	05/31/2011	09/20/2012	478	FDA	2333
Dinutuximab	Neuroblastoma	Mab	04/11/2014	03/10/2015	333	12/05/2013	08/14/2015	617	FDA	157
Durvalumab	Differing Indications	Mab	10/13/2016	05/01/2017	200[AA]	09/01/2017	09/21/2018	385	FDA	508
Elotuzumab	Multiple Myeloma	Mab	06/29/2015	11/30/2015	154	07/03/2015	05/11/2016	313	FDA	163
Encorafenib	Melanoma	KI	06/30/2017	06/27/2018	362	07/28/2017	09/19/2018	418	FDA	84
Entrectinib	NTRK gene fusion	KI	12/18/2018	08/15/2019	240[AA]	01/07/2019	07/31/2020	571[CA]	FDA	351

© 2022 Lythgoe MP et al. *JAMA Network Open.*

Enzalutamide	Prostate Cancer	Endo	05/22/2012	08/31/2012	101	06/26/2012	06/21/2013	360	FDA	294
Eribulin	Breast Cancer	CC	03/30/2010	11/15/2010	230	03/30/2010	03/17/2011	352	FDA	122
Gilteritinib	Acute Myeloid Leukemia	KI	03/29/2018	11/28/2018	244	02/07/2019	10/24/2019	259	FDA	330
Glasdegib	Acute Myeloid Leukemia	Other	04/27/2018	11/21/2018	208	04/29/2019	06/26/2020	424	FDA	583
Ibrutinib	Mantle Cell Lymphoma	KI	06/28/2013	11/13/2013	138[AA]	10/29/2013	10/21/2014	357	FDA	342
Idelalisib	Various	KI	09/11/2013	07/23/2014	315[AA]	10/28/2013	09/18/2014	325	FDA	57
Inotuzumab ozogamicin	Acute Lymphocytic Leukemia	ADC	12/20/2016	08/17/2017	240	04/14/2016	06/28/2017	440	EMA	50
Ipilimumab	Melanoma	Mab	06/25/2010	03/25/2011	273	05/05/2010	07/13/2011	434	FDA	110
Ixazomib	Multiple Myeloma	Other	07/10/2015	11/20/2015	133	07/30/2015	11/21/2016	480[CA]	FDA	367
Larotrectinib	NTRK gene fusion	KI	03/26/2018	11/26/2018	245[AA]	08/24/2018	09/19/2019	391[CA]	FDA	297
Lenvatinib	Papillary Thyroid Cancer	KI	08/14/2014	02/13/2015	183	08/15/2014	05/28/2015	286	FDA	104
Lorlatinib	Non-Small Cell Lung Cancer	KI	12/05/2017	11/02/2018	332[AA]	01/09/2018	05/06/2019	482[CA]	FDA	185

© 2022 Lythgoe MP et al. *JAMA Network Open.*

Lutetium Lu 177 dotatate	Gastro-entero-pancreatic Neuroendocrine Tumours	Other	04/28/2016	01/26/2018	638	04/26/2016	09/26/2017	518	EMA	122
Midostaurin	Acute Myeloid Leukemia	KI	08/29/2016	04/28/2017	242	07/22/2016	09/18/2017	423	FDA	143
Mogamulizumab	Cutaneous T-cell Lymphoma	Mab	10/04/2017	08/08/2018	308	10/06/2017	11/22/2018	412	FDA	106
Moxetumomab pasudotox	Hairy Cell Leukemia	ADC	01/29/2018	09/13/2018	227	11/22/2019	02/08/2021	444	FDA	879
Necitumumab	Non-Small Cell Lung Cancer	Mab	12/02/2014	11/24/2015	357	12/01/2014	02/15/2016	441	FDA	83
Neratinib	Breast Cancer	KI	07/19/2016	07/17/2017	363	06/23/2016	08/31/2018	799	FDA	410
Niraparib	Ovarian Cancer	Other	10/31/2016	03/27/2017	147	10/04/2016	11/16/2017	408	FDA	234
Nivolumab	Melanoma	Mab	07/30/2014	12/22/2014	145[AA]	09/02/2014	06/19/2015	290	FDA	179
Obinutuzumab	Chronic Lymphocytic Leukemia	Mab	04/22/2013	11/01/2013	193	04/25/2013	07/22/2014	453	FDA	263
Ofatumumab	Chronic Lymphocytic Leukemia	Mab	01/30/2009	10/26/2009	269[AA]	02/05/2009	04/19/2010	438[CA]	FDA	175
Olaparib	Ovarian Cancer	Other	02/03/2014	12/19/2014	319[AA]	09/03/2013	12/16/2014	469	EMA	3

© 2022 Lythgoe MP et al. *JAMA Network Open.*

Olaratumab	Soft Tissue Sarcoma	Mab	02/24/2016	10/19/2016	238[AA]	01/29/2016	11/09/2016	285[CA]	FDA	21
Osimertinib	Non-Small Cell Lung Cancer	KI	06/05/2015	11/13/2015	161[AA]	06/05/2015	02/01/2016	241[CA]	FDA	80
Palbociclib	Breast Cancer	KI	08/13/2014	02/03/2015	174[AA]	07/30/2015	11/09/2016	468	FDA	645
Panobinostat	Multiple Myeloma	Other	03/24/2014	02/23/2015	336[AA]	05/05/2014	08/28/2015	480	FDA	186
Pazopanib	Renal Cell Carcinoma	KI	12/19/2008	19/10/2009	304	02/27/2009	06/14/2010	472[CA]	FDA	238
Pembrolizumab	Melanoma	Mab	02/27/2014	09/04/2014	189[AA]	06/04/2014	07/17/2015	408	FDA	316
Pertuzumab	Breast Cancer	Mab	12/08/2011	06/08/2012	183	12/01/2011	03/04/2013	459	FDA	269
Polatuzumab vedotin	Diffuse Large B-cell Lymphoma	ADC	12/19/2018	06/10/2019	173[AA]	12/20/2018	01/16/2020	392[CA]	FDA	220
Pomalidomide	Multiple Myeloma	Other	04/10/2012	02/08/2013	304[AA]	05/29/2012	08/05/2013	433	FDA	178
Ponatinib	Various	KI	09/27/2012	12/14/2012	78[AA]	08/30/2012	07/01/2013	305	FDA	199
Radium-223	Prostate Cancer	Other	12/14/2012	05/15/2013	152	12/12/2012	11/13/2013	336	FDA	182
Ramucirumab	Gastric Cancer	Mab	08/23/2013	04/21/2014	241	08/23/2013	12/19/2014	483	FDA	242
Regorafenib	Colorectal Cancer	KI	04/27/2012	09/27/2012	153	05/03/2012	08/26/2013	480	FDA	333

© 2022 Lythgoe MP et al. *JAMA Network Open.*

Ribociclib	Breast Cancer	KI	08/29/2016	03/13/2017	196	09/05/2016	08/22/2017	351	FDA	162
Rucaparib	Ovarian Cancer	Other	06/23/2016	12/19/2016	179[AA]	11/01/2016	05/23/2018	568[CA]	FDA	520
Ruxolitinib	Myelofibrosis	KI	06/03/2011	11/16/2011	166	06/01/2011	08/23/2012	449	FDA	281
Siltuximab	Castleman's Disease	Mab	08/30/2013	04/23/2014	236	08/30/2013	05/22/2014	266	FDA	29
Sipuleucel-T	Prostate Cancer	Other	10/30/2009	04/29/2010	181	12/30/2011	09/06/2013	616	FDA	1226
Sonidegib	Basel Cell Carcinoma	Other	09/26/2014	07/24/2015	301	05/05/2014	08/14/2015	466	FDA	21
Tagraxofusp-erzs	Blastic plasmacytoid dendritic cell neoplasm	Mab	06/21/2018	12/21/2018	183	01/07/2019	01/07/2021	731	FDA	748
Talazoparib	Breast Cancer	Other	04/06/2018	10/16/2018	193	04/27/2018	06/20/2019	419	FDA	247
Talimogene laherparepvec	Melanoma	Other	07/28/2014	10/27/2015	456	08/28/2014	12/16/2015	475	FDA	50
Tisagenlecleucel	Diffuse Large B-cell Lymphoma	Other	02/02/2017	08/30/2017	209	11/02/2017	08/22/2018	293	FDA	357
Trabectedin	Differing Indications	CC	11/24/2014	10/23/2015	333	07/27/2006	09/17/2007	417	EMA	2958
Trametinib	Melanoma	KI	08/03/2012	05/29/2013	299	02/07/2013	06/30/2014	508	FDA	397

© 2022 Lythgoe MP et al. *JAMA Network Open.*

Trastuzumab deruxtecan-nxki	Breast Cancer	ADC	08/29/2019	12/20/2019	113[AA]	05/22/2020	01/18/2021	241[CA]	FDA	395
Trifluridine and tipiracil	Colorectal Cancer	CC	12/19/2014	09/22/2015	277	02/27/2015	04/25/2016	423	FDA	216
Vandetanib	Medullary Thyroid Cancer	KI	07/07/2010	04/06/2011	273	09/01/2010	02/16/2012	533[CA]	FDA	316
Vemurafenib	Melanoma	KI	04/28/2011	08/17/2011	111	05/04/2011	02/17/2012	289	FDA	184
Venetoclax	Chronic Lymphocytic Leukemia	Other	10/29/2015	04/11/2016	165[AA]	11/13/2015	12/04/2016	387[CA]	FDA	237
Vismodegib	Basel Cell Carcinoma	Other	09/08/2011	01/30/2012	144	12/01/2011	07/12/2013	589[CA]	FDA	529
Ziv-aflibercept	Colorectal Cancer	Mab	02/03/2012	08/03/2012	182	11/24/2011	02/01/2013	435	FDA	182

Key: KI – Kinase Inhibitor**; Endo** – Endocrine therapy**; ADC** – Antibody Drug Conjugate**; Mab** – Monoclonal Antibody**; CC** – Cytotoxic Therapies**; Other** – other unclassified therapy (see supplementary table 2)

AA – Accelerated Approval
CA – Conditional Approval

Notes: Author analysis of data from online Food and Drug Administration (FDA;http://www.fda.gov) and European Medicines Agency (EMA; http://www.ema.europa.eu)

© 2022 Lythgoe MP et al. *JAMA Network Open.*

eTable 2: Oncology therapy approvals classified as 'other' by mechanism of action

Drug	Mechanism of Action
Axicabtagene ciloleucel	CD19-directed genetically modified autologous T cell immunotherapy
Carfilzomib	Proteasome inhibitor
Decitabine	Nucleoside metabolic inhibitor
Ixazomib	Proteasome inhibitor
Lutetium Lu 177 dotatate	Radiolabelled somatostatin analogue
Niraparib	Poly(ADP-ribose) polymerase (PARP) inhibitor
Olaparib	Poly(ADP-ribose) polymerase (PARP) inhibitor
Panobinostat	Histone deacetylase inhibitor
Pomalidomide	Thalidomide analogue
Radium Ra-223 Dichloride	Alpha particle-emitting radioactive therapeutic agent
Rucaparib	Poly(ADP-ribose) polymerase (PARP) inhibitor
Sipuleucel-T	Autologous cellular immunotherapy
Sonidegib	Hedgehog pathway inhibitor
Tagraxofusp	CD123-directed cytotoxin
Talazoparib	Poly (ADP-ribose) polymerase (PARP) inhibitor
Talimogene laherparepvec	Genetically modified oncolytic viral therapy
Tisagenlecleucel	CD19-directed genetically modified autologous T-cell immunotherapy
Venetoclax	BCL-2 inhibitor
Vismodegib	Hedgehog pathway inhibitor

© 2022 Lythgoe MP et al. *JAMA Network Open.*

eFigure 1: Waterfall plot showing time difference (in days) between FDA new oncology approval and publication of pivotal trial results

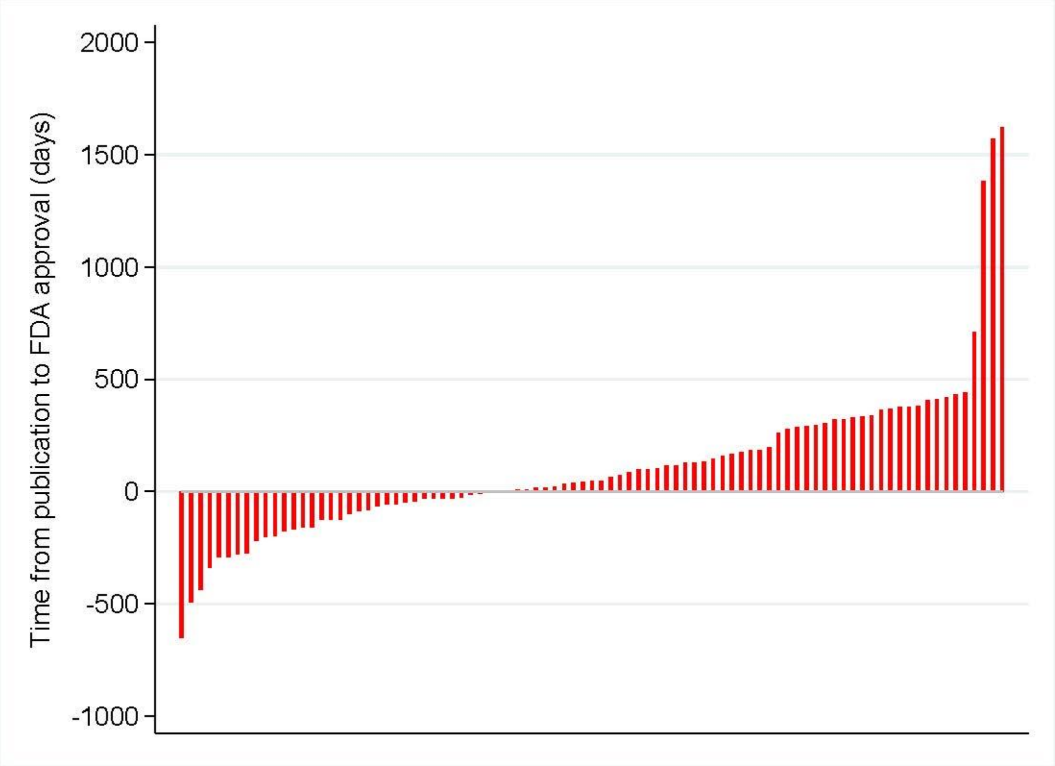

Note: Each vertical line represents one drug approval. A positive time difference indicates approval was after pivotal trial publication, and a negative time difference that approval was before pivotal trial publication

© 2022 Lythgoe MP et al. *JAMA Network Open.*

eFigure 2: Waterfall plot showing time difference (in days) between EMA new oncology approval and publication of pivotal trial results

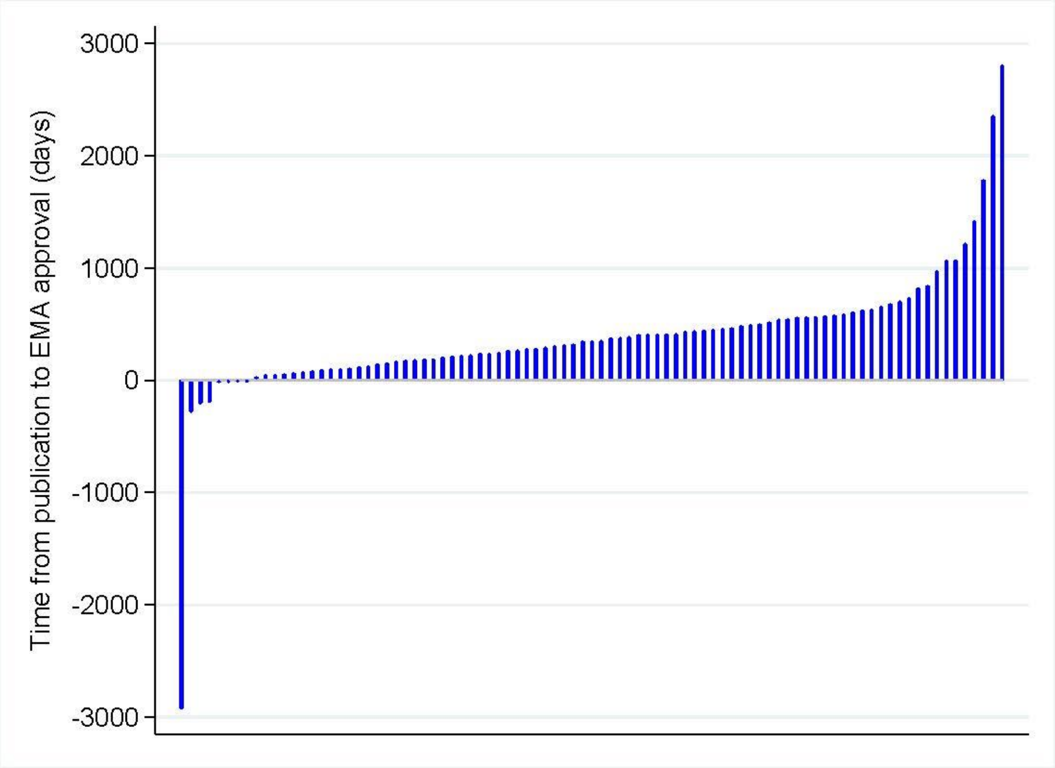

Note: Each vertical line represents one drug approval. A positive time difference indicates approval was after pivotal trial publication, and a negative time difference that approval was before pivotal trial publication

© 2022 Lythgoe MP et al. *JAMA Network Open.*
